# Mechanisms underlying the predictive power of high skeletal muscle uptake of FDG in amyotrophic lateral sclerosis

**DOI:** 10.1186/s13550-020-00666-6

**Published:** 2020-07-07

**Authors:** Cecilia Marini, Vanessa Cossu, Tiziana Bonifacino, Matteo Bauckneht, Carola Torazza, Silvia Bruno, Patrizia Castellani, Silvia Ravera, Marco Milanese, Consuelo Venturi, Sebastiano Carlone, Patrizia Piccioli, Laura Emionite, Silvia Morbelli, Anna Maria Orengo, Maria Isabella Donegani, Alberto Miceli, Stefano Raffa, Stefano Marra, Alessio Signori, Katia Cortese, Federica Grillo, Roberto Fiocca, Giambattista Bonanno, Gianmario Sambuceti

**Affiliations:** 1 CNR Institute of Molecular Bioimaging and Physiology (IBFM), Milano, Italy; 2 Nuclear Medicine, IRCCS Ospedale Policlinico San Martino, Largo Benzi 10, 16132 Genova, Italy; 3 Department of Health Sciences, University of Genoa, Genova, Italy; 4 Department of Pharmacy, Section of Pharmacology and Toxicology and Center of Excellence for Biomedical Research, University of Genoa, Genova, Italy; 5 Department of Experimental Medicine, Human Anatomy, University of Genoa, Genova, Italy; 6 Cell Biology, IRCCS Ospedale Policlinico San Martino, Genova, Italy; 7 Animal Facility, IRCCS Ospedale Policlinico San Martino, Genova, Italy; 8 Department of Surgical Sciences and Integrated Diagnostics, Pathology Unit, University of Genoa, Genova, Italy; 9 Pharmacology and Toxicology, IRCCS Ospedale Policlinico San Martino, Genova, Italy

**Keywords:** Amyotrophic lateral sclerosis, SOD1^G93A^ mouse model, [18F]*-*Fluorodeoxyglucose, Skeletal muscle, Oxidative stress, Endoplasmic reticulum

## Abstract

**Background:**

We recently reported that enhanced [18F]-fluorodeoxyglucose (FDG) uptake in skeletal muscles predicts disease aggressiveness in patients with amyotrophic lateral sclerosis (ALS). The present experimental study aimed to assess whether this predictive potential reflects the link between FDG uptake and redox stress that has been previously reported in different tissues and disease models.

**Methods:**

The study included 15 SOD1^G93A^ mice (as experimental ALS model) and 15 wildtype mice (around 120 days old). Mice were submitted to micro-PET imaging. Enzymatic pathways and response to oxidative stress were evaluated in harvested quadriceps and hearts by biochemical, immunohistochemical, and immunofluorescence analysis. Colocalization between the endoplasmic reticulum (ER) and the fluorescent FDG analog 2-[N-(7-nitrobenz-2-oxa-1,3-diazol-4-yl)amino]-2-deoxyglucose (2-NBDG) was performed in fresh skeletal muscle sections. Finally, mitochondrial ultrastructure and bioenergetics were evaluated in harvested quadriceps and hearts.

**Results:**

FDG retention was significantly higher in hindlimb skeletal muscles of symptomatic SOD1^G93A^ mice with respect to control ones. This difference was not explained by any acceleration in glucose degradation through glycolysis or cytosolic pentose phosphate pathway (PPP). Similarly, it was independent of inflammatory infiltration. Rather, the high FDG retention in SOD1^G93A^ skeletal muscle was associated with an accelerated generation of reactive oxygen species. This redox stress selectively involved the ER and the local PPP triggered by hexose-6P-dehydrogenase. ER involvement was confirmed by the colocalization of the 2-NBDG with a vital ER tracker. The oxidative damage in transgenic skeletal muscle was associated with a severe impairment in the crosstalk between ER and mitochondria combined with alterations in mitochondrial ultrastructure and fusion/fission balance. The expected respiratory damage was confirmed by a deceleration in ATP synthesis and oxygen consumption rate. These same abnormalities were represented to a markedly lower degree in the myocardium, as a sample of non-voluntary striated muscle.

**Conclusion:**

Skeletal muscle of SOD1^G93A^ mice reproduces the increased FDG uptake observed in ALS patients. This finding reflects the selective activation of the ER-PPP in response to significant redox stress associated with alterations of mitochondrial ultrastructure, networking, and connection with the ER itself. This scenario is less severe in cardiomyocytes suggesting a relevant role for either communication with synaptic plaque or contraction dynamics.

## Background

Amyotrophic lateral sclerosis (ALS) is a neurodegenerative disease of upper and lower motor neurons leading to a severe impairment in the interplay between the nervous motor chain and the skeletal muscle, eventually resulting in progressive paralysis and death [1–4]. Although the underlying mechanisms remain uncertain, several studies suggested a relevant role for the imbalance between reactive oxygen species (ROS) generation and detoxification [5, 6] both in the central nervous system and skeletal muscles. Nevertheless, the absence of methods able to evaluate the redox stress and the antioxidant responses in vivo so far prevented to define the relevance of this alteration in ALS progression.

We previously reported a predictive power of spinal cord [18F]-fluorodeoxyglucose (FDG) uptake computationally extracted from PET/CT images of ALS patients [7]. The apparent metabolic activation of this nervous district faced an opposite pattern in the brain cortex that showed a generalized reduction in tracer uptake [8]. By contrast, it extended to the psoas muscles of the same patients, suggesting a link between the metabolic pattern of the second motoneuron and its effector [9].

FDG uptake is commonly considered an index of glucose consumption. Nevertheless, several studies recently challenged this classical assumption and reported a direct and strict link between tracer retention and the activation of a specific pentose phosphate pathway (PPP) triggered by the “omnivore” enzyme hexose-6P-dehydrogenase (H6PD) within the endoplasmic reticulum (ER) of cancer cells [10, 11], neurons and astrocytes [12], cardiomyocytes [13] and, more importantly, in skeletal muscles [14]. A major function of PPP is the reduction of NADP^+^ to NADPH to fuel the glutathione-dependent antioxidant responses. Accordingly, the present study aimed to verify whether this kinetic model accounts for the capability of FDG uptake to track the redox mechanisms underlying ALS skeletal muscle damage, at least in SOD1^G93A^ experimental model. This analysis was complemented with the evaluation of the myocardium, in order to verify whether the metabolic reprogramming selectively affects the motor chain or, rather, it reflects a systemic phenomenon involving all striated muscles regardless of their connection with the second motor neuron.

## Material and methods

### Animal models

All experiments were carried out in accordance with the guidelines established by the European Communities Council (Directive 114 2010/63/EU of September 22, 2010) and with Italian DL n.26/2014 and were approved by the local Ethical Committee and by the Italian Ministry of Health (Authorization No. 97/2017-PR). The study included thirty 118/127-day-old mice (Jackson Laboratories, Bar Harbor, ME, USA): 15 B6SJL-Tg (SOD1*G93A)1Gur mice expressing high copy number of mutant human SOD1 with a Gly93Ala substitution (SOD1^G93A^) and 15 background-matched B6SJL wildtype mice, considered as the “control group” [15]. All transgenic mice were bred in the animal facility of the Pharmacology and Toxicology Unit, Department of Pharmacy, University of Genoa (Italy), and identified by analyzing tissue extracts from tail tips, as previously described [16]. SOD1^G93A^ mice were studied at a late phase of disease and synchronized by motor impairment determination, defined by our validated method [17]. Age-matched wildtype mice were used as controls. Both female and male mice were used in the experiments; sexes were balanced in each experimental group to avoid bias due to sex-related intrinsic differences. Animals were housed at constant temperature (22 ± 1 °C) and relative humidity (50%) with a regular 12-h light cycle (light 7 AM–7 PM). Food (type 4RF21 standard diet; Mucedola, Settimo Milanese, Italy) and water were freely available. All efforts were made to minimize animal suffering and to use only the number of animals necessary to produce reliable results. All mice were euthanized by cervical dislocation.

### Experimental micro-PET imaging

Sample size of mice submitted to micro-PET imaging was defined based on the hypothesis of a 40% increase of skeletal muscle FDG uptake induced by ALS, coupled with an expected variation coefficient of tracer retention in this same tissue approaching 16%. These features were entered into a freely available calculator (http://www.rad.jhmi.edu/jeng/javarad/samplesize/#references) that estimated sample size to five mice per group as proposed by Eng et al. [18]. All animals were fasted for 6 h, weighted, and anesthetized with intra-peritoneal ketamine (100 mg/Kg) and xylazine (10 mg/kg). Serum glucose level was tested and FDG (3–4 MBq) was injected through a tail vein. Forty minutes later, mice underwent a 10-min static acquisition in a dedicated micro-PET system (Albira, Bruker, USA) whose dual ring configuration allows the acquisition of the whole mouse body. After its completion, mice were immediately euthanized by cervical dislocation.

### Ex vivo extraction fraction of FDG and glucose

Three mice per group were sacrificed; quadriceps and hearts were harvested to analyze ex vivo FDG uptake and glucose consumption. The quadriceps muscle side was randomly selected. Ex vivo FDG uptake of quadriceps and hearts was evaluated using the Ligand-Tracer White® instrument (Ridgeview, Uppsala, SE) according to our previously validated procedure [12, 14, 19]. Briefly, the device consists of a beta-emission detector and a rotating platform harboring a standard Petri dish. The rotation axis is inclined at 30° from the vertical, so that the organ alternates its position from the nadir (for incubation) to the zenith (for counting) every minute. Slices (300 μm thick) of quadriceps or hearts were stuck in the outer ring of a Petri dish with octyl-cyanoacrylate (Dermabond, Ethicon, US) and covered with 3 mL solution collected from an input vial containing 4 mL of Dulbecco’s modified Eagle’s medium (DMEM) containing glucose and FDG at the concentration of 5.5 mM (1 g/L) and 2 MBq/mL, respectively. Time-activity curves were thus obtained by subtracting decay-corrected background counting rate from the corresponding target value, as previously described [12, 14, 19, 20]. After 40 min, an aliquot of 0.5 mL was sampled both from the input vial and from the Petri dish (output) to measure the radioactivity concentration using a dose calibrator with an activity resolution < 10 KBq (Capintec CRC55).

Fractional FDG uptake was thus calculated as:

$$ \mathrm{Fractional}\ \mathrm{FDG}\ \mathrm{uptake}=\frac{A_{\mathrm{input}}-{A}_{\mathrm{output}}}{A_{\mathrm{input}}} $$

where *A*_input_ and *A*_output_ represent activity (MBq) in the surnatant before and after exposure to the muscle specimen, respectively.

After the measurement of FDG radioactivity concentration, the medium aliquots were appropriately stored for glucose assay. Glucose consumption was measured as:

$$ \mathrm{Fractional}\ \mathrm{Glucose}\ \mathrm{intake}=\left[\frac{C_{\mathrm{input}}-{C}_{\mathrm{output}}}{C_{\mathrm{input}}}\right] $$

where *C*_input_ and *C*_output_ represent the corresponding mM concentrations in the surnatant before and after exposure to the muscle specimen, respectively. Medium glucose concentration was assayed following the reduction of NADP, at 340 nm, using the following solutions: 100 mM Tris-HCl pH 7.4, 2 mM MgCl2, 2 mM ATP, and 4 mg HK/G6PD (Sigma-Aldrich).

### Sample preparation for biochemical analyses

Three dedicated quadriceps and three hearts of each group were homogenized with a Potter-Elvehjem in 1 mL of homogenization buffer (0.25 M sucrose, 0.15 M KCl, 1 mM EDTA, 10 Mm Tris-HCl pH 7.4). An aliquot of obtained homogenate was immediately frozen at − 80 °C while the remaining sample was immediately used for mitochondria isolation. Briefly, the homogenate was centrifuged at 800×*g* for 10 min to precipitate nuclei and cellular debris. The supernatant collected was centrifuged for 15 min at 12,000×*g*. The pellet obtained, containing mitochondria, was resuspended in a solution buffer (0.225 M sucrose, 0.075 M mannitol, 10 mM Tris-HCl pH 7.4, 1 mM EDTA), centrifuged for 10 min at 1,000×*g* and washed by centrifugation at 12,000×*g* for 15 min [21]. Protein concentration was tested by Bradford analysis [22].

### Oxygen consumption and ATP synthesis assay in isolated mitochondria

Oxygen consumption was measured in fresh mitochondria by means of an amperometric oxygen electrode (Microrespiration, Unisense A/S, Århus, Denmark) in a closed chamber (volume 1.7 ml) magnetically stirred, at room temperature. For each fresh sample, 25 μg of proteins were incubated in the respiration buffer composed of 120 mM KCl, 2 mM MgCl_2_, 1 mM KH_2_PO_4_, 50 mM Tris HCl, pH 7.4, and 25 μg/ml ampicillin. The following substrates were used: 5 mM pyruvate + 2.5 mM malate to stimulate complexes I-III-IV [23].

A luciferin/luciferase chemiluminescence method was used to measure ATP synthase in fresh mitochondria. For each sample, 25 μg of proteins were incubated for 10 min at 37 °C in a medium that contained: 10 mM Tris/HCl (pH 7.4), 100 mM KCl, 1 mM EGTA, 2.5 mM EDTA, 5 mM MgCl_2_, 5 mM KH_2_PO_4_, 0.6 mM Ouabain, and Ampicillin (25 μg/ml). The same substrates described for the oxymetric analysis were used. After 10 min of incubation, 0.1 mM ADP was added, to induce ATP synthesis, which was measured by means of a luminometer (Glomax 20/20 Luminometer, Promega Italia, Milano, Italy). ATP standard solutions in the concentration range 10^−9^–10^−5^ M were used for calibration [24].

### Enzymatic assay

Enzymatic assays were performed using frozen homogenates of three quadriceps and three hearts of each group. Enzymatic assays were performed in a double beam spectrophotometer (UNICAM UV2, Analytical S.n.c., Italy) [10–14].

Hexokinase (HK), hexose-6-phostate dehydrogenase (H6PD), and glucose-6-phosphate dehydrogenase (G6PD) activities were assayed following reduction of NADP, at 340 nm. Phosphofructokinase (PFK) activity was assayed following oxidation of NADH, at 340 nm.

The following assays solutions were used: HK: Tris-HCl-pH 7.4-100 mM (TRIS7.4), MgCl_2_ 2 mM, glucose 200 mM, ATP 1 mM, NADP 0.5 mM, and 2 UI G6PD (Sigma-Aldrich); H6PD: TRIS7.4, 2DG6P 10 mM, NADP 0.5 mM; G6PD: TRIS7.4, G6P 10 mM, and NADP 0.5 mM; and PFK: Tris-HCl pH 8 100 mM, MgCl_2_ 2 mM, KCl 5 mM, fructose-6-phosphate 2 mM, ATP 1 mM, phosphoenolpyruvate (PEP) 0.5 mM, NADH 0.2 mM, and 2 IU pyruvate kinase (PK)/4 IU lactate dehydrogenase (LDH) (Sigma-Aldrich).

Glutathione reductase (GR) activity and NADPH/NADP ratio were evaluated spectrophotometrically, at 405 and 450 nm, respectively, using GR Assay Kit (Abcam: ab83461) and NADP-NADPH Assay Kit (Abcam: ab65349), following the manufacturer’s instructions.

To assess lipid peroxidation, malondialdehyde (MDA) levels were evaluated, by the thiobarbituric acid reactive substances (TBARS) assay, with minor modifications [13, 25]. The activity assay of the complex I was measured on 50 μg of total protein as previously described [10, 12, 26].

### Western blot analysis

Western blot (WB) experiments were performed according to the standard procedure. Frozen homogenates were prepared from the quadriceps of three mice per group and were sonicated twice for 10 s in ice, with a break of 30 s. Denaturing electrophoresis (SDS-PAGE) was performed using Mini PROTEAN TGX Precast gels (Bio Rad). For each sample, 25 μg of total protein were loaded. Run was performed at 4 °C, at 70 mA for each gel, for 30–40 min. Running buffer contained 0.05 M Tris (pH 8.0), 0.4 M glycine, 1.8 mM EDTA, and 0.1% SDS. Electrophoretically separated samples were transferred onto nitrocellulose (NC) membranes by electroblotting, at 400 mA for 1 h in Tris-glycine buffer (50 mM Tris and 380 mM glycine) plus 20% (v/v) methanol, at 4 °C. NC membranes were incubated with the specific antibodies diluted in Tris Buffer Saline + 0.15% tween (TBST). We tested the following antibodies: anti-mitofusin 2 (Mfn2, ThermoFisher: PA5 72811, 1:1000), anti-dynamin-1-like protein (Drp1, ThermoFisher: PA5 34768, 1:2000), anti-calnexin (ThermoFisher: MA3-027, 1:500), and anti-GAPDH (Cell Signaling: #5174, 1:1000). After extensive washing with 0.15% TBST, 1 h of incubation with secondary horseradish peroxidase-conjugated Abs diluted in TBST (1:10,000 for anti-mouse and anti-rabbit antibodies) was performed.

### Bioimaging by confocal microscopy

The experiments were performed on dedicated fresh skeletal muscle sections from three SOD1^G93A^ and three control mice, promptly after excision. The sections were gently stretched to reduce thickness without affecting the viability of tissue cells (verified by exclusion of the non-permeable dye DAPI in parallel samples) and immediately incubated at 37 °C for 10 min with the fluorescent probes 2-[N-(7-nitrobenz-2-oxa-1,3-diazol-4-yl)amino]-2-deoxyglucose (2-NBDG) (50 μM ) and ER-Tracker^TM^ Red (1 μM), both from Molecular Probes (InVitrogen, Eugene, OR). Nuclei were stained with the cell-permeable dye Hoechst 33342 (10 μM). Images were obtained using the SP2-AOBS confocal microscope (Leica Microsystems, Mannheim, Germany).

### Immunofluorescence and immunohistochemical analysis

Soon after sacrifice, 3 half-hearts and 3 quadriceps were harvested from controls and from SOD1^G93A^ mice, embedded in OCT and snap-frozen in precooled isopentane. Six-micrometer-thick serial cryostat sections were obtained.

For the immunofluorescence analysis, immediately after cutting, the sections were fixed for 10 min in cold acetone, followed by incubation with MitoTracker® Red (Molecular Probes), at 10 nM for 20 min at 37 °C. For reactive oxygen species (ROS) staining, serial sections from the same samples were fixed for 10 min in acetone, followed by incubation with 10 μM 2′,7′-dichlorofluorescein diacetate (H_2_DCFDA; Molecular Probes) for 30 min and a wash in PBS.

For immunohistochemical staining of skeletal muscles infiltrated macrophages the following antibodies were used: rat anti-mouse CD206 (AbD Serotec, 1:200, 5 μg/ml) to detect macrophages M2, rat anti-mouse CD86 clone PO.3 (Millipore, 1:100, 5 μg/ml) to detect macrophages M1, and rat anti-mouse CD11b (Novus Biologicals, 1:200, 5 μg/ml).

### Ultrastructural analysis

Three quadriceps and three hearts per group were dissected and fixed in 0.1 M cacodylate buffer containing 2.5% glutaraldehyde (Electron Microscopy Science, Hatfield, PA, USA), for 2 h at room temperature. Samples were postfixed in osmium tetroxide for 2 h and 1% uranyl acetate for 2 h. Samples were next dehydrated through a graded ethanol series and propylene oxide and embedded in epoxy resin (Poly-Bed; Polysciences, Inc., Warrington, PA, USA) overnight at 42 °C and 2 days at 60 °C. Ultrathin sections (50 nm) were counterstained with 5% uranyl acetate and lead citrate and observed with transmission electron microscope (TEM) HT7880, Hitachi, Japan.

### Image analysis

Micro-PET images were reconstructed using a maximum-likelihood expectation-maximization (MLEM) algorithm and were qualitatively inspected. Images were analyzed using the routine of a commercial software (PMOD, Zurich, CH). Two nuclear medicine physicians, unaware of model nature (transgenic or wildtype), drew volumes of interest (VOIs) on the left and right hindlimb skeletal muscles and on the metabolically active left ventricular myocardium to measure the mean standardized uptake value (SUV) according to the formula:

$$ \mathrm{SUV}=\frac{\left[\mathrm{FDG}\right]\times \mathrm{body}\ \mathrm{weight}}{\mathrm{Injected}\ \mathrm{activity}} $$

where [FDG] indicates average FDG concentration in kBq/ml within any given VOI and bodyweight is expressed in kilograms and injected activity in MBq. Skeletal muscle and myocardial FDG concentration were divided by the corresponding value in the blood pool to obtain the SUV ratio (SUVr). For WB analysis, the signal was acquired with Alliance 6.7 WL 20 M (UVITEC, Cambridge, UK), and UV1D software (UVITEC). Densitometry analysis was performed using the dedicated routine of ImageJ software (ImageJ Version 2.0.0-rc-65/1.51 s). For colocalization analysis, six randomly selected fields were analyzed in three independent samples harvested from either SOD1^G93A^ and control mice. Original unadjusted and uncorrected images were processed using ImageJ software for the evaluation of colocalization, which was expressed as the percentage of above-background pixels in 2-NBDG images that overlapped above-background pixels in ER images, with background threshold set by the Costes’ method [27].

For immunofluorescence analysis, images were acquired by the Fluoview FV500 software and the fluorescence was quantified using the same ImageJ software. Immunohistochemical images were acquired with Leica DM RX microscopy and were analyzed using Scion Image software [28].

Finally, for ultrastructural analysis, digital images were acquired with Megaview 3 camera (EMSIS GmbH, Germany).

### Statistical analysis

Data are presented as mean ± standard deviation. Two-tailed Student *t* test for unpaired data was used. Levene’s test for equality of variances was used to test the homogeneity of variances of the two groups. When homogeneity was not satisfied, the Welch-Satterthwaite method was used to adjust the *p* value for this assumption violation. Statistical significance was considered for *p* < 0.05. In all analyses, *p* value was complemented by the evaluation of standardized difference between the two means estimated by Cohen’s *d* index. A complete description of these evaluations is reported in Supplementary Table 1. Statistical analyses were performed using SPSS software 26.0 (Chicago, IL, USA).

## Results

### SOD1^G93A^ mutation and FDG uptake in skeletal muscle

**Fig. 1:**
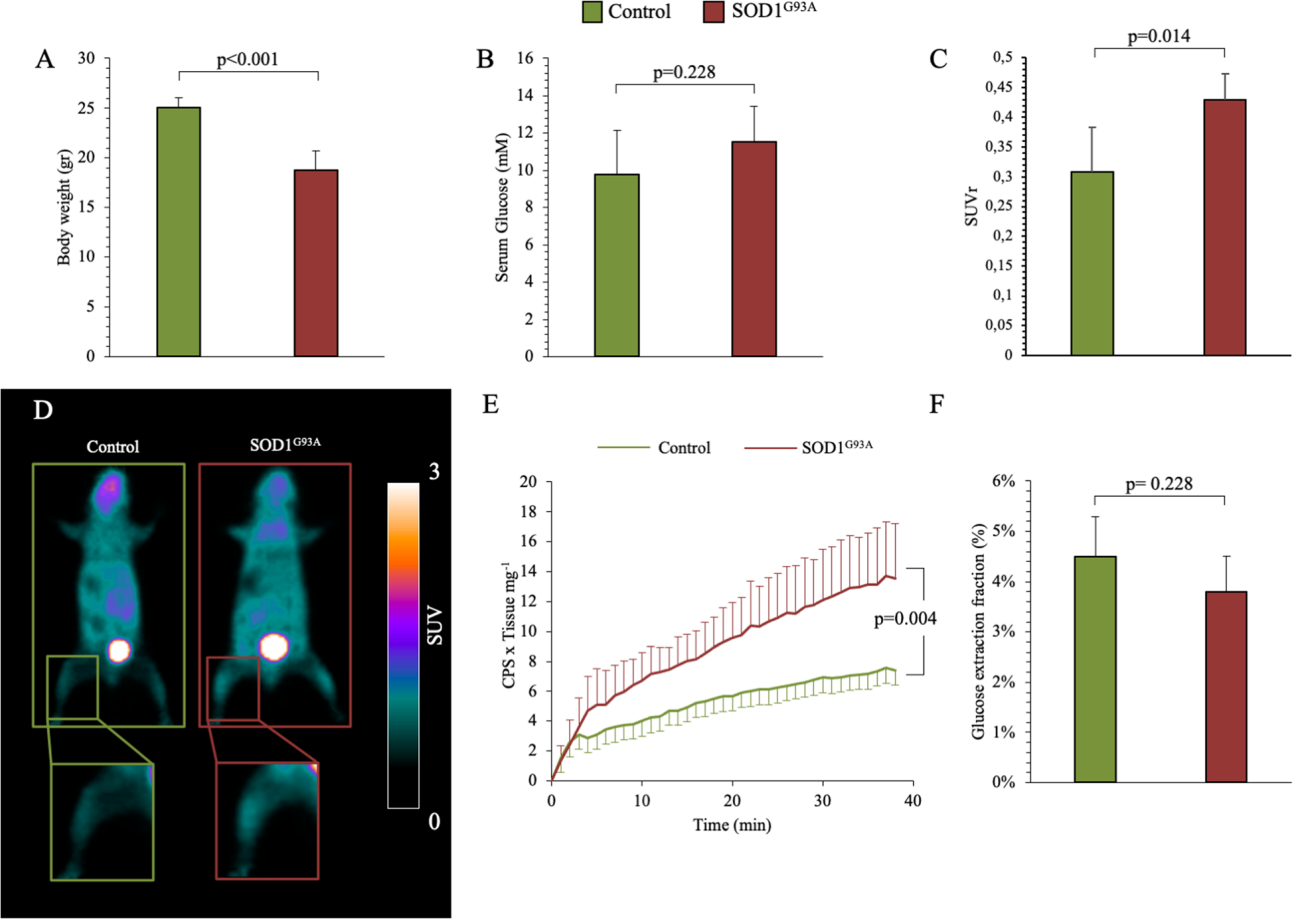
In vivo and ex vivo effects of SOD1^G93A^ mutation in skeletal muscle. **a**, **b** Bodyweight and serum glucose levels in controls (green column) and SOD1^G93A^ mice (red column). **c** Average of FDG retention expressed as standardized uptake value ratio (SUVr) in control (green column) and SOD1^G93A^ skeletal muscle (red column). **d** Representative hindlimb SUV of control and SOD1^G93A^ mice. **e** Average time-course of “ex-vivo” radioactivity expressed as a fraction of the FDG dose measured by the Ligand-Tracer White® device, in quadriceps harvested from control (green line) and SOD1^G93A^ mouse (red line). **f** Ex vivo glucose consumption of harvested quadriceps from control (green column) and SOD^1G93A^ mouse (red column). Data are expressed as mean ± SD; **a**–**d***n* = 5 for each group; **e**, **f***n* = 3 for each group. Student *t* test for unpaired data was used for statistical evaluation

At the time of micro-PET imaging (around 120 days after birth), body weight was significantly lower in SOD1^G93A^ mice compared to controls (Fig. 1a) while no differences were observed in serum glucose levels between the two groups (Fig. 1b). Hindlimb SUVr was significantly higher in SOD1^G93A^ mice with respect to controls (0.43 ± 0.04 vs. 0.31 ± 0.07 in SOD1^G93A^ and control groups, respectively, *p* = 0.014, Fig. 1c, d). This difference also applied to rough SUV data (0.44 ± 0.09 vs. 0.33 ± 0.03 in mutated and control mice, respectively, *p* = 0.042, Supplementary Table 2).

This finding was reproduced ex vivo, since FDG accumulation was higher in quadriceps harvested from SOD1^G93A^ mice compared to the corresponding muscles harvested from wildtype littermates (Fig. 1e). In agreement with our hypothesis, the increase in tracer uptake was not paralleled by any difference in glucose extraction that was similar in control and SOD1^G93A^ quadriceps (Fig. 1f).

### Inflammatory markers

**Fig. 2:**
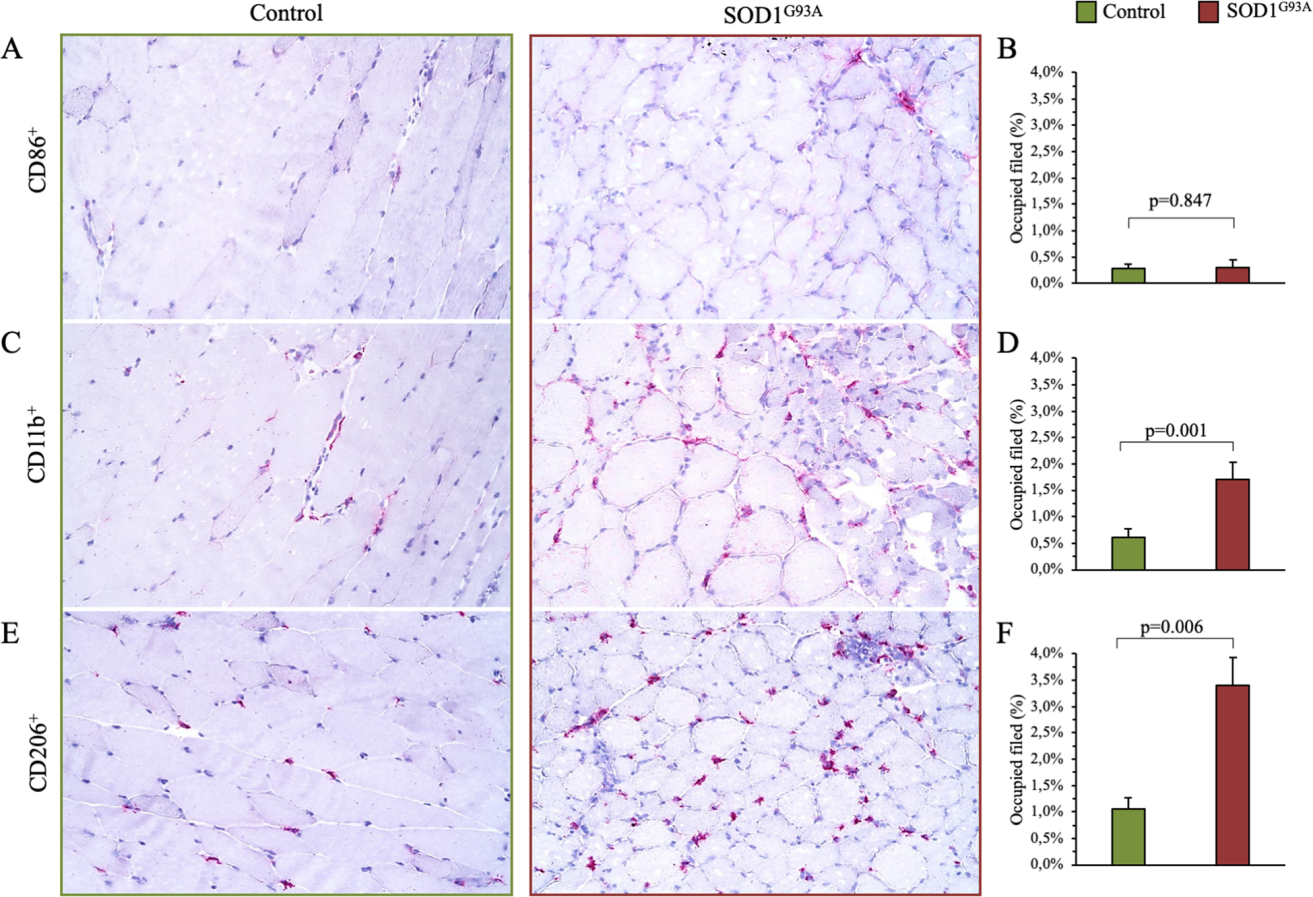
Effect of SOD1^G93A^ in quadriceps inflammatory infiltration. **a**, **c**, **e** Immunohistochemical representative images of CD86, CD11b, and CD206 expression, in controls (green edge line) and SOD1^G93A^ (red edge line) quadriceps. **b**, **d**, **f** Quantitative representation of the CD86^+^, CD11b^+^, and CD206^+^ signal, expressed as a percent of the image field occupied by the specific cell staining. Data are expressed as mean ± SD, *n* samples = 3 for each group. Student *t* test for unpaired data was used for statistical evaluation

The increased tracer uptake of skeletal muscles was not fully explained by inflammatory infiltrates. Indeed, immunohistochemical analysis showed that CD86^+^ positive cells, responsible for the high tracer retention in inflammation, were scarcely represented and superimposable in both controls and SOD1^G93A^ skeletal muscle (Fig. 2a, b). By contrast, CD11b^+^ and CD206^+^ positive cells were more represented in SOD1^G93A^ quadriceps (Fig. 2c and d, and e and f, respectively). However, the prevalence of both CD11b^+^ and CD206^+^ was too low (< 4% of the imaged field) to justify the increased FDG accumulation in transgenic skeletal muscle.

### The activity of rate-limiting enzymes of glucose degradation in skeletal muscle of SOD1^G93A^ mice

**Fig. 3:**
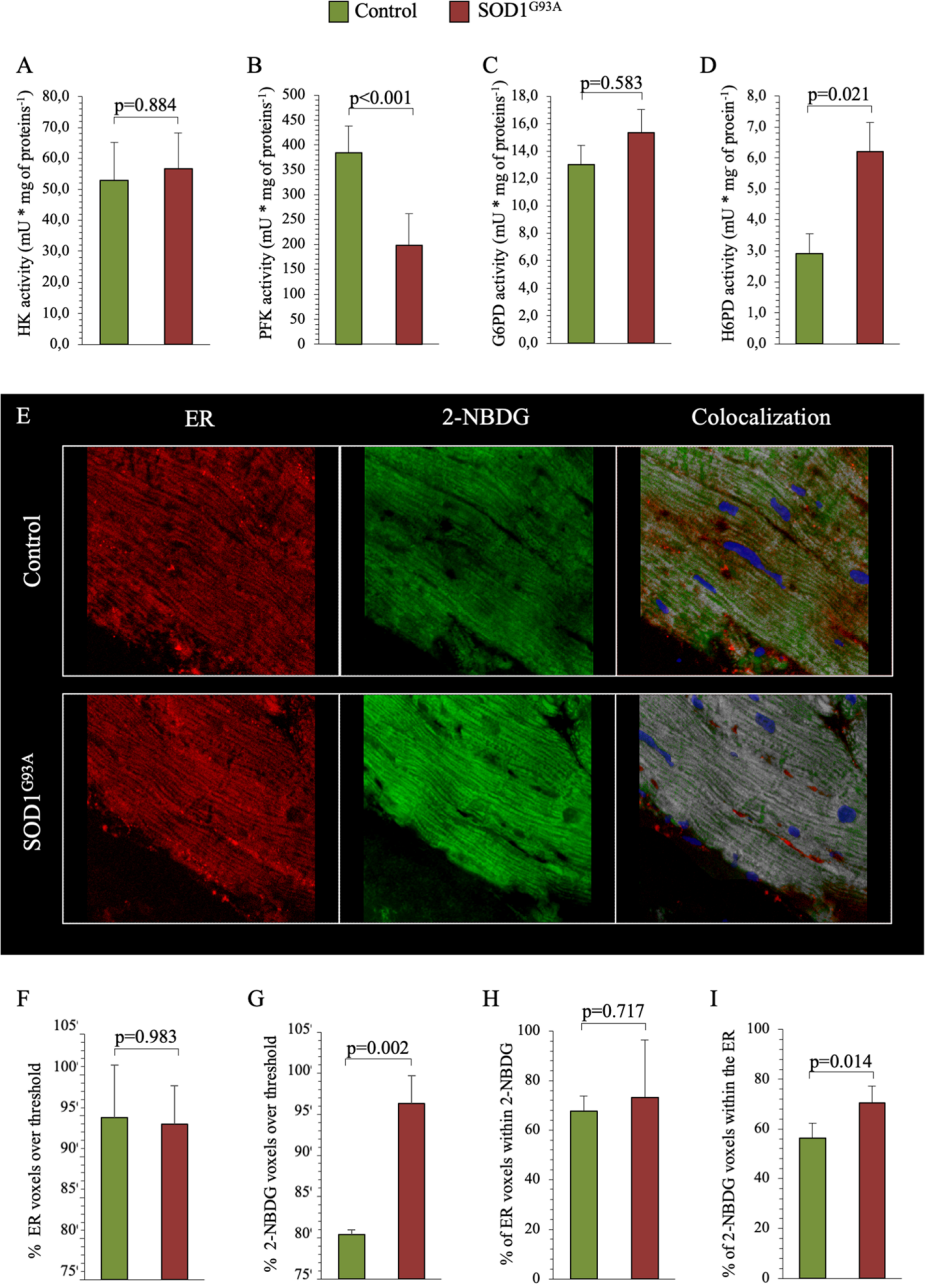
Effect of SOD1^G93A^ mutation on enzymatic pathway and colocalization of 2-NBDG and ER signals in harvested quadriceps. **a**–**d** Respective activities of HK, PFK, G6PD, and H6PD in control (green column) and SOD1^G93A^ groups (red column) of quadriceps homogenate. **e** Immunofluorescence representative images of ER (red fluorescence), 2-NBDG (green fluorescence), and colocalization staining signals (% of 2-NBDG voxels within the ER staining—white) in control and SOD1^G93A^ quadriceps. Blue color reports the Hoechst 33342 signal. **f**, **g** Percent voxels over threshold of ER and 2-NBDG, respectively, in control and SOD1^G93A^ quadriceps. **h** Percent of ER voxels within 2-NBDG staining. **i** Percent of 2-NBDG voxels within ER staining. Data are expressed as mean ± SD, *n* = 3 for each group. Student *t* test for unpaired data was used for statistical evaluation

The effect of SOD1^G93A^ mutation on FDG uptake in skeletal muscle was not explained by the response of enzymes regulating cytosolic glucose catabolism. Indeed, HK catalytic function was not affected by SOD1^G93A^ mutation (Fig. 3a), while PFK activity was significantly reduced in transgenic muscles (Fig. 3b). An analogous consideration also applies to the cytosolic PPP, since G6PD catalytic function was superimposable in SOD1^G93A^ and control littermates (Fig. 3c). By contrast, the enhanced tracer uptake matched the behavior of H6PD, whose catalytic function was significantly increased in SOD1^G93A^ skeletal muscle with respect to controls (Fig. 3d).

### Colocalization of 2-NBDG and ER signals in harvested quadriceps

The agreement between FDG uptake and H6PD activity suggested the activation of ER PPP in SOD1^G93A^ skeletal muscles. This hypothesis was confirmed by confocal microscopy: ER signal intensity was similar in muscle samples obtained from either group (Fig. 3e, f). By contrast and in agreement with the analysis of FDG retention, 2-NBDG fluorescence was significantly higher in SOD1^G93A^ quadriceps than in their wildtype littermates (Fig. 3e, g). At colocalization analysis, the fraction of ER voxels (volumetric picture elements) containing 2-NBDG was similar in both control and mutated skeletal muscle (Fig. 3e, h). However, the percentage of 2-NBDG voxels included within the ER was significantly higher in transgenic mice (Fig. 3e, i) indicating that SOD1^G93A^ mutation increases the mobilization of this glucose analog from the cytosol to the ER.

### Antioxidant response and oxidative stress

**Fig. 4:**
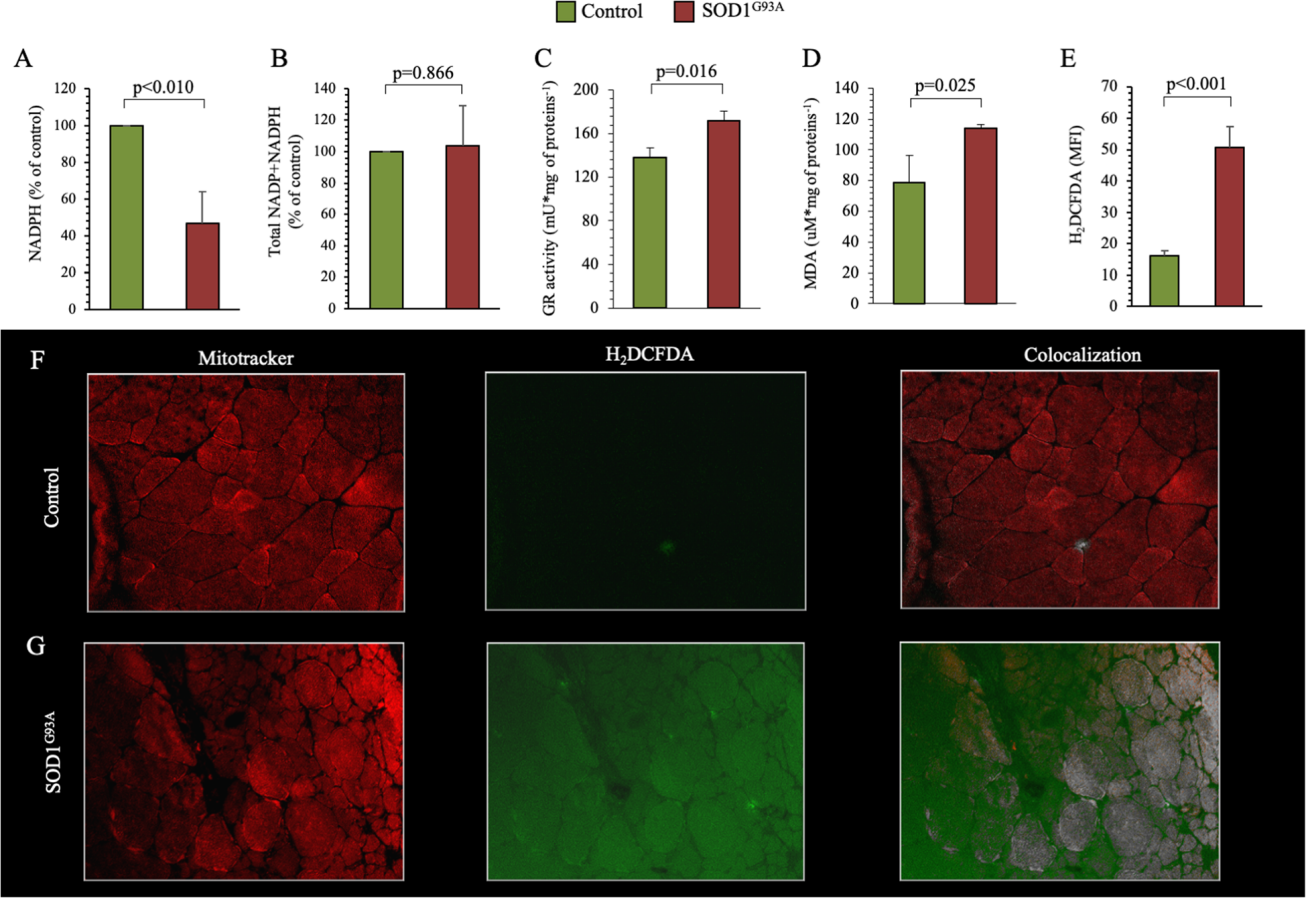
Antioxidant response and oxidative stress in SOD1^G93A^ mutation. **a**, **b** NADPH levels and total NADP+NADPH content in control (green column) and SOD1^G93A^ (red column) quadricep homogenates represented as percent of control. **c** GR activity and **d** MDA content in control (green column) and SOD1^G93A^ (red column) quadriceps. **e** Mean fluorescence index (MFI) of H_2_DCFDA in control and SOD1^G93A^ quadriceps muscles. **f** Immunofluorescence representative images of MitoTracker® (as specific mitochondria staining—red fluorescence), H_2_DCFDA (as specific ROS staining—green fluorescence), and colocalization staining signals, in control and SOD1^G93A^ quadriceps. Data are expressed as mean ± SD, *n* = 3 for each group. Student *t* test for unpaired data was used for statistical evaluation

Altogether, obtained data suggest an acceleration of ER PPP, which should be associated with an increment of the NADPH production. Conversely, NADPH appeared significantly lower in transgenic quadriceps with respect to their wildtype littermates (Fig. 4a), despite a superimposable content of cofactor equivalents (NADP and NADPH) (Fig. 4b). However, this apparent discrepancy was explained by the higher GR activity in SOD1^G93A^ quadriceps with respect to controls (Fig. 4c), since this enzyme uses NADPH as a cofactor.

Despite the increment of the enzymatic activities involved in the antioxidant defenses, SOD1^G93A^ mutation was associated with a significant increment of lipid peroxidation and ROS level in SOD1^G93A^ skeletal muscle in comparison to the wildtype littermates, as shown by the combined increase in MDA levels (Fig. 4d) and H_2_DCFDA-associated fluorescence (Fig. 4f). Intriguingly, H_2_DCFDA fluorescence was also found in sample regions devoid of MitoTracker® signal, suggesting a contribution of mitochondria with impaired membrane polarization to ROS generation.

### Mitochondrial ultrastructure and bioenergetic evaluation in skeletal muscle

**Fig. 5:**
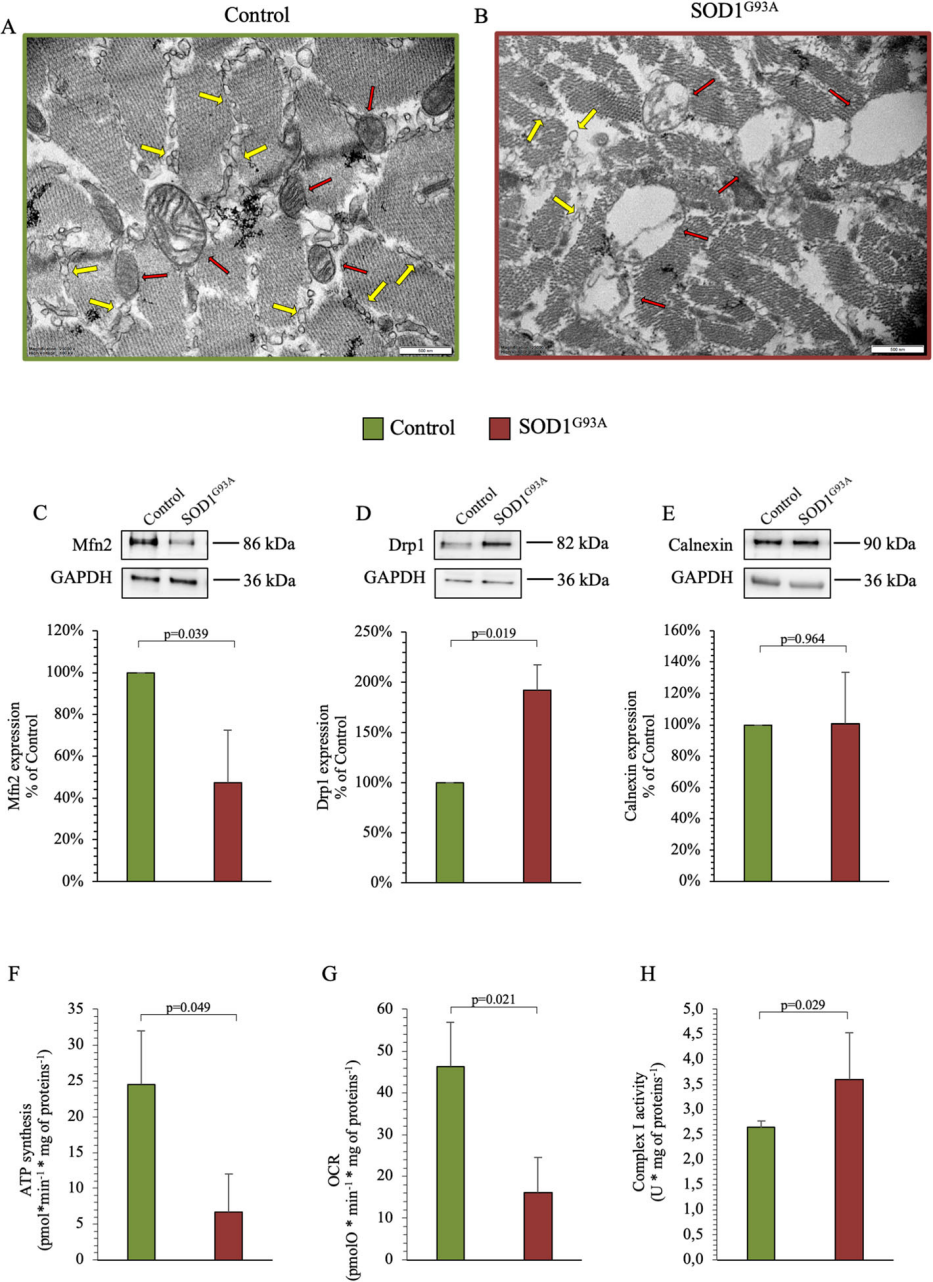
Mitochondrial/ER ultrastructure and bioenergetics activity in skeletal muscles. **a**, **b** Electron microscopy representative images of control and SOD1^G93A^ quadriceps (*n* = 3). In both panels, red arrows show mitochondria while yellow arrows show ER ultrastructural profiles. **c**–**e** Western blot analysis and relative densitometry quantitative analyses of Mfn2, Drp1, and Calnexin in the two groups. **f**, **g** ATP synthesis and oxygen consumption rate stimulated by pyruvate/malate in control (green column) and SOD1^G93A^ (red column) of isolated mitochondria. **h** Complex I activity in isolated mitochondria form control (green column) and SOD1^G93A^ muscles (red column) studied by the FeCN reduction in the presence of NADH. Data are expressed as mean ± SD, *n* = 3 for each group. Student *t* test for unpaired data was used for statistical evaluation

In the control group, normal mitochondria with well-defined cristae and membrane structure were seen in sub-sarcolemma and intermyofibrillar regions (Fig. 5a). By contrast, SOD1^G93A^ quadriceps showed an evident alteration of mitochondrial inner cristae, suggesting an enhanced oxidative stress. In addition, transgenic skeletal muscles showed abnormal mitochondrial networking coupled with a severe impairment in their shape (Fig. 5b) as well as with a profound disruption in the mitochondrion-ER connection.

Consequently, we evaluated the main regulator of the mitochondrial fusion/fission machinery, by western blot analysis: Mfn2, a mitochondria-associated membrane (MAM) protein that promotes mitochondrial fusion, and Drp1, a GTPase involved in mitochondrial fission. SOD1^G93A^ skeletal muscle showed a significant decrease in Mfn2 protein amount (Fig. 5c) opposite to the increase of Drp1content with respect to control (Fig. 5d). Despite a significant alteration in mitochondrial fusion/fission, calnexin abundance did not change under SOD1^G93A^ mutation (Fig. 5e).

These observations matched the functional alterations observed in quadriceps mitochondria. Indeed, ATP synthesis was significantly lower in mutated isolated mitochondria of SOD1^G93A^ muscles with respect to control ones (Fig. 5f). This effect agreed with the reduction in oxygen consumption rate through complexes I-III-IV triggered by pyruvate-malate (Fig. 5g). Nevertheless, this response disagreed with the selective enhancement of complex I activity that was observed in mutated muscles when studied by the FeCN reduction in the presence of NADH (Fig. 5h) that, in turn, justifies the increase in ROS production.

### Evaluation of glucose metabolism and redox status in the myocardium

Since the myocardium is a striated involuntary muscle independent of cholinergic neuromuscular synapses, we here investigated, for the first time, whether its function can be affected by the SOD1^G93A^ mutation.

**Fig. 6:**
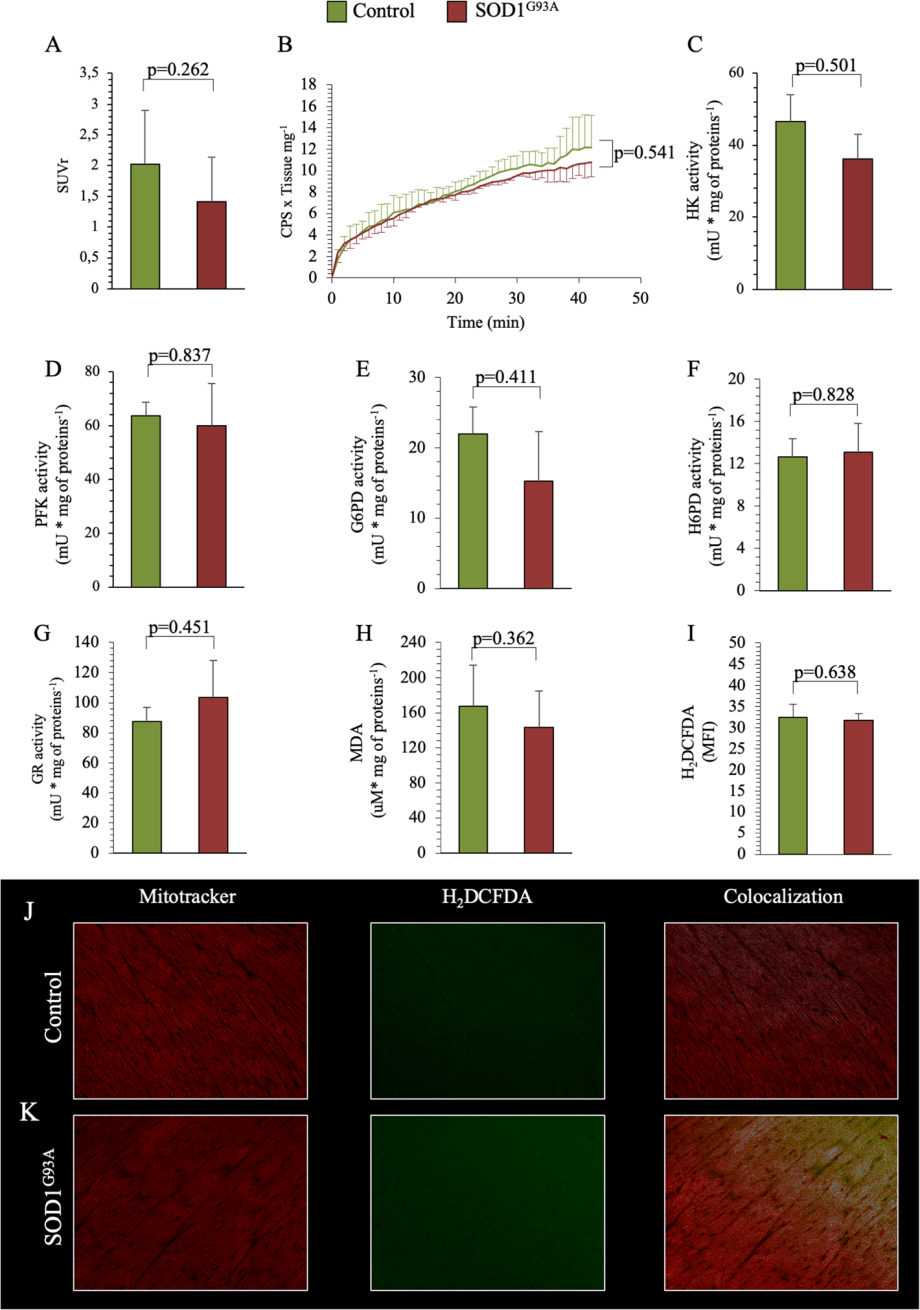
Evaluation of glucose metabolism and redox status in the cardiac myocardium of SOD1^G93A^ and control mice. **a** Average of FDG retention expressed as standardized uptake value ratio (SUVr) in control (green column) and SOD1^G93A^ myocardium (red column). Data are expressed as mean ± SD, *n* = 5 for each group. **b** Average time-course of “ex-vivo” radioactivity expressed as a fraction of the FDG dose, measured by the Ligand-Tracer White® apparatus, in myocardium harvested from control (green line) and SOD1^G93A^ mice (red line). **c**–**f** Myocardial catalytic activities of HK, PFK, G6PD, H6PD, and GR (**g**) in control (green column) and SOD1^G93A^ (red column). **h** MDA levels and **i** MFI H_2_DCFDA in control and SOD1^G93A^ experimental groups. **j**, **k** Immunofluorescence representative images of Mitotracker® (as specific mitochondria staining—red fluorescence), H_2_DCFDA (as specific ROS staining—green fluorescence), and colocalization staining signals, in control and SOD1^G93A^ myocardium. Data are expressed as mean ± SD, *n* = 3 for each group. Student *t* test for unpaired data was used for statistical evaluation

In vivo experiments did not show any differences in myocardium SUV and SUVr between control and mutated mice (Suppl Table 2 and Fig. 6a, respectively). Similarly, ex vivo SOD1^G93A^ myocardium FDG kinetics accumulation was superimposable with respect to control littermates (Fig. 6b).

The catalytic function of the main enzymes involved in cytosolic glucose catabolism (HK, PFK, and G6PD) was similar between control and mutated myocardium (Fig. 6c–e). Moreover, SOD1^G93A^ did not affect the activity of the reticular enzyme H6PD (Fig. 6f). In agreement with these data, the antioxidant response evaluated by GR catalytic function was not affected by SOD1^G93A^ mutation (Fig. 6g) as well as the indexes of oxidative stress evaluating by MDA levels (Fig. 6h) and H_2_DCFDA-associated fluorescence (Fig. 6j). Finally, the relatively less evident damage with respect to skeletal muscle was confirmed by the virtual absence of inflammatory infiltrates in myocardial specimens.

### Inflammatory infiltrates, mitochondrial function, and ultrastructure in the myocardium

Effect of SOD1^G93A^ mutation on inflammatory infiltrates was less evident in the myocardium with respect to skeletal muscle. Indeed at immunohistochemical analysis, CD86^+^ and CD11b^+^ cells were virtually not represented in both groups (Suppl Figure 1, Panels A-D). On the other hand, evidence of CD206^+^ was similar in control and SOD1^G93A^ (Suppl Figure 1, E-F).

**Fig. 7:**
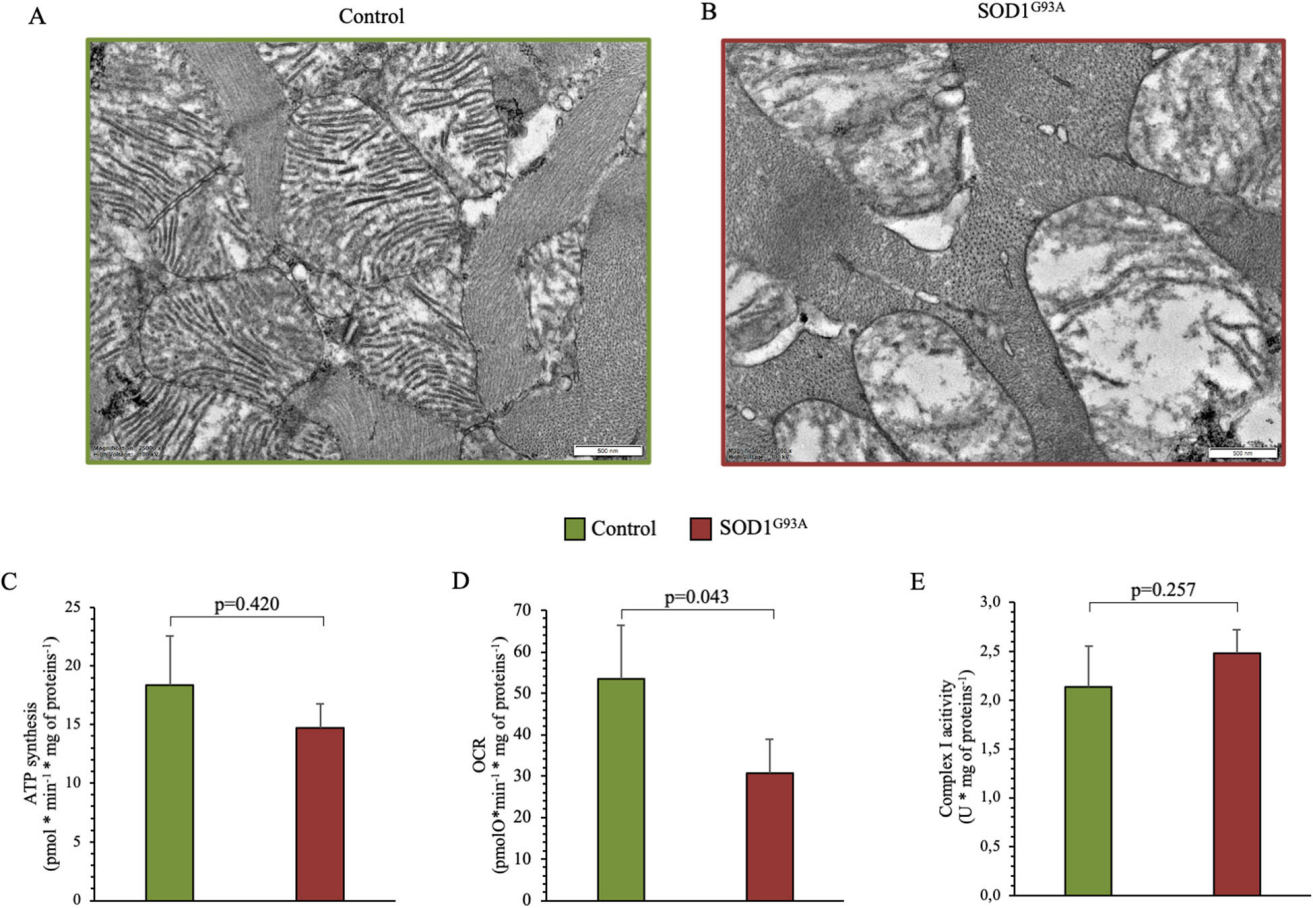
Mitochondrial ultrastructure and bioenergetics activity in cardiac myocardium of SOD1^G93A^ and control mice. **a**, **b** Electron microscopy representative images of control SOD1^G93A^ myocardium. **c**, **d** ATP synthesis and oxygen consumption rate stimulated by pyruvate/malate in control (green column) and SOD1^G93A^ (red column) of isolated mitochondria. **e** Complex I activity in isolated mitochondria of control (green column) and of mutated muscles (red column) studied by the FeCN reduction in the presence of NADH. Data are expressed as mean ± SD, *n* = 3 for each group. Student *t* test for unpaired data was used for statistical evaluation

Ultrastructural analysis confirmed the relatively lower involvement of the heart. With respect to skeletal muscle, mitochondria of mutated myocardium showed relatively less evident alterations in shape and in number of cristae that appeared disorganized and relatively less represented (Fig. 7a, b) in transgenic hearts. In agreement with the lower degree of ultrastructural impairment, isolated mitochondria from SOD1^G93A^ myocardium showed a slight, but not significant, decrease in ATP synthesis associated with a significant reduction in OCR without any alteration in complex I activity (Fig. 7c–e).

## Discussion

The present study confirms that the increased skeletal muscle FDG uptake observed in ALS patients also occurs in the SOD1^G93A^ experimental model [9]. The increase in FDG accumulation rate was observed in living mice, under fasting conditions and in the presence of normal glucose levels. It was not caused by a physical activity, because mice were under anesthesia during the tracer distribution time. Moreover, it persisted ex vivo, under the incubation in a medium devoid of both hormonal signals and competing metabolites. The enhanced tracer avidity of skeletal muscles of transgenic SOD1^G93A^ mice was not explained by inflammation. Indeed, the most FDG-avid inflammatory cells, CD86^+^ macrophages [29–33], were scarcely represented in SOD1^G93A^ quadriceps muscle with a similar abundance with respect to control mice. Actually, CD11b^+^ and CD206^+^ cells were significantly more abundant in SOD1^G93A^ quadriceps. However, they were only barely represented both in control and SOD1^G93A^ quadriceps; thus, this difference was too low to account for the evident and diffuse increase of FDG uptake observed in mutated mice [34–36]. Finally, the high tracer avidity of SOD1^G93A^ muscles also extended to the FDG analog 2-NBDG, whose fluorescence was diffusely increased in all myocytes. Thus, the present findings indicate that the increased FDG uptake reflects a metabolic shift of skeletal muscles.

Classical models consider an increased FDG uptake as the sign of an accelerated glucose consumption. Accordingly, our findings apparently disagree with recent evidence reporting a significant role of impaired glucose metabolism in ALS disease progression. Indeed, glycolytic flux had been found to be impaired in both the spinal cord [37] and gastrocnemius muscles [38] of different ALS experimental models while interventions able to increase glucose utilization [39, 40] were proven to provide a therapeutic benefit on disease progression. Similarly, the increased FDG retention conflicts with the response of PFK and G6PD activity, induced by SOD1^G93A^ mutation, that would rather suggest a deceleration in glycolytic flux combined with a constant rate of cytosolic PPP. However, the divergent response of tracer retention and glycolytic flux to SOD1^G93A^ mutation closely agrees with a series of previous observations [10–14] documenting a relative independence between FDG uptake and glucose consumption. According to this evidence, tracer accumulation rate largely reflects the activation of H6PD catalytic function within the ER as also confirmed by the selective localization of the FDG fluorescent analog, 2-NBDG, within the ER of transgenic muscles.

H6PD is the acknowledged trigger of the first two PPP reactions within the ER [41]. As for its cytosolic counterpart, the main role of this reticular pathway is the NADP^+^ reduction to NADPH to fuel both bio-reductive syntheses [42] and glutathione-dependent antioxidant responses [43]. In the present study, SOD1^G93A^ quadriceps showed increased levels of both lipid peroxidation and ROS production, as suggested by the high levels of MDA and H_2_DCFDA-associated fluorescence coupled with the enhanced GR activity and the decreased NADPH/NADP ratio. On the other hand, the present data also indicate that the antioxidant response was not sufficient to counterbalance the oxidative stress production, as demonstrated by the lipid peroxidation accumulation in the SOD1^G93A^ muscle. More interestingly, the evident response of H6PD, coupled with the relative invariance of G6PD catalytic function, configures the ER as a selective target of redox stress in transgenic muscles.

The high vulnerability of the ER to oxidative damage agrees with the strictly functional and physical connection of this organelle with the mitochondria whose electron transport chain is recognized as the main cellular ROS source [44–47]. Similarly, it matches the double target and contributor role attributed to the same ER in cell redox control [48], with glucose metabolism involved in all the three canonical branches of unfolded protein response, namely the inositol-requiring kinase 1 (IRE1) [49], protein kinase R-like ER kinase (PERK) [50], and activating transcription factor 6 (ATF6) [51] pathways.

In agreement with these assumptions and confirming the evidence in the literature for both patients and mouse experimental models [52–54], TEM showed a dramatic alteration in mitochondrial networking, coupled with a severe impairment in their shape and further associated with the loss of cristae in transgenic mice. This ultrastructural evidence confirms previous studies in ALS reporting a profound disruption in the mitochondrion-ER relationship [55], and thus in the main regulator of the fission/fusion machinery, used by all post-mitotic tissues, including skeletal muscle, to adapt mitochondrial morphology and function to cell bioenergetic requirements [56, 57]. This dynamic balance of mitochondrial location, size, and number is regulated by a series of GTPases; among these, Mfn2 promotes mitochondrial fusion as opposed to Drp1 that instead favors mitochondrial fission. The relevance of this equilibrium has been recently recognized by Tezze et al., who reported a significant reduction of fusion and fission proteins in atrophic muscles of elderly sedentary people [58]. Similarly, Drp1 protein level has been found to decrease during aging in cardiac and skeletal muscles of normal mice [59]. In the present study, the SOD1^G93A^ mutation was associated with significantly decreased levels of Mfn2, facing an increased abundance of its opponent Drp1. Accordingly, the present data indicate that the muscular damage, associated with the SOD1^G93A^ mutation, recognizes different mechanisms with respect to the aging process [60], although the present data do not permit to elucidate whether this consideration also extends to ALS patients.

The profound disruption of mitochondrial morphology and dynamics nicely agrees with the impairment in oxygen consumption rate and ATP synthesis that was observed in transgenic mice in agreement with previous literature [61–63]. However, despite a reduction of oxidative phosphorylation metabolism, the activity of complex I was higher in SOD1^G93A^ muscle in comparison to the control sample, in agreement with previous observations [64]. This increment can explain the increase of ROS production, despite the reduction of aerobic activity, since complex I is considered the main oxidative stress producer of the electron transport chain [65]. Moreover, the maintenance of complex I activity suggests that the impairment of oxidative phosphorylation activity is principally associated with the loss of cristae organization and mitochondrial morphology and dynamics.

Finally, the simultaneous evaluation of the heart documented a markedly less evident alteration in cardiomyocytes of transgenic mice in all evaluated immune-histochemical, functional, and ultrastructural indexes. To the best of our knowledge, this evaluation is the first attempt to compare myocardial and skeletal muscle response to experimental ALS. The evidence of a cardiomyocyte impairment, though less severe or slower in its progression with respect to skeletal muscle, fits with previous studies documenting a relatively high incidence of left ventricular dysfunction in patients with long-lasting motor neuron disease [66]. It also agrees with previous evidence about a wide distribution of TDP-43 aggregates in post-mortem specimens of skeletal and cardiac muscle in ALS patients [67].

## Conclusion

In conclusion, our study documents that the increased FDG uptake observed in ALS patients is reproduced in SOD1^G93A^ mice and is not associated with any shift in cytosolic glucose metabolism. Rather, it reflects the selective activation of the ER-PPP triggered by the omnivore enzyme H6PD in response to a significant redox stress at least partially caused by an alteration of mitochondrial structure, networking, and connection with the ER itself. This series of events selectively involves skeletal muscles and is markedly less severe in cardiomyocytes suggesting a relevant role of either communication with a synaptic plaque or contraction dynamics. The present data may justify further research to identify methods interrogating the ER response to redox damage as a tool able to discover the earliest subclinical signs of this lethal neurodegenerative disease and possibly a new useful and non-invasive prognostic marker for ALS.

## Supplementary information

**Additional file 1: Supplementary Table 1.** Statistical parameters of Independent Samples Test

**Additional file 2: Supplementary Table 2.** PET imaging data

**Additional file 3.** Supplementary Materials

**Additional file 4.** Suppl Figure

## Data Availability

The datasets generated during and/or analyzed during the current study are available from the corresponding author on reasonable request.

## Abbreviations

ALS: Amyotrophic lateral sclerosis
SUV: Average standardized uptake value
FDG: [18F]-Fluorodeoxyglucose
ROS: Reactive oxygen species
PPP: Pentose phosphate pathway
G6PD: Glucose-6-phosphate dehydrogenase
H6PD: Hexose-6P-dehydrogenase
ER: Endoplasmic reticulum
PFK: Phosphofructokinase
GR: Glutathione reductase
MDA: Malondialdehyde
HK: Hexokinase
2-NBDG: 2-[N-(7-Nitrobenz-2-oxa-1,3-diazol-4-yl)amino]-2-deoxyglucose
H_2_DCFDA: 2′,7′-Dichlorofluorescein diacetate
Mfn2: Mitofusin 2
Drp1: Dynamin-1-like protein
