# Supplementary material for: Mechanisms underlying the predictive power of high skeletal muscle uptake of FDG in amyotrophic lateral sclerosis
Source: EJNMMI Res. 2020 Jul 7;10:76. doi: 10.1186/s13550-020-00666-6 (PMC7340686; doi:10.1186/s13550-020-00666-6)
Supplement: Supplementary file 1 — Additional file 1: Supplementary Table 1. Statistical parameters of Independent Samples Test [file 13550_2020_666_MOESM1_ESM.pdf]

Supplementary Table 1: Statistical parameters of Independent Samples Test

		Levene's Test for Homoscedasticity		t-test for Equality of Means			Cohen's d
		F	*Sig.*	*t*	*df*	*Sig. (2-tailed)*	
**Body weight**	**Equal variances assumed**	**5.00**	**0.056**	**6.53**	**8**	**0.000**	**4.13**
	Equal variances not assumed			6.53	6.012	0.001	
**Serum glucose level**	**Equal variances assumed**	**0.01**	**0.915**	**-1.30**	**8**	**0.228**	**0.82**
	Equal variances not assumed			-1.30	7.624	0.23	
**SM VOIs**	**Equal variances assumed**	**3.72**	**0.090**	**0.10**	**8**	**0.921**	**0.10**
	Equal variances not assumed			0.10	4.457	0.923	
**Myocardium VOIs**	**Equal variances assumed**	**5.01**	**0.056**	**0.45**	**8**	**0.666**	**0.44**
	Equal variances not assumed			0.45	5.573	0.671	
**SM SUVr**	**Equal variances assumed**	**2.93**	**0.125**	**-3.13**	**8**	**0.014**	**1.98**
	Equal variances not assumed			-3.13	6.397	0.019	
**Myocardial SUVr**	**Equal variances assumed**	**0.69**	**0.429**	**1.21**	**8**	**0.262**	**0.76**
	Equal variances not assumed			1.21	7.730	0.263	
**SM SUV**	Equal variances assumed	18.56	0.003	-2.76	8	0.025	**1.75**
	**Equal variances not assumed**			**-2.76**	**4.783**	**0.042**	
**Myocardial SUV**	**Equal variances assumed**	**0.47**	**0.511**	**0.97**	**8**	**0.361**	**0.61**
	Equal variances not assumed			0.97	7.929	0.361	
**Blood pool FDG concentration**	**Equal variances assumed**	**2.80**	**0.133**	**0.00**	**8**	**0.228**	**1.30**
	Equal variances not assumed			-1.14	6.027	0.24	
**SM FDG concentration**	**Equal variances assumed**	**4.30**	**0.072**	**-2.41**	**8**	**0.043**	**2.40**
	Equal variances not assumed			-2.41	4.245	0.07	
**Myocardial FDG concentration**	**Equal variances assumed**	**0.62**	**0.455**	**-0.10**	**8**	**0.922**	**0.10**
	Equal variances not assumed			-0.10	6.283	0.922	
**Ex vivo SM FDG uptake**	**Equal variances assumed**	**2.07**	**0.224**	**-6.16**	**4**	**0.004**	**5.03**
	Equal variances not assumed			-6.16	2.557	0.014	
**Ex vivo Heart FDG uptake**	**Equal variances assumed**	**1.11**	**0.352**	**0.67**	**4**	**0.541**	**0.67**
	Equal variances not assumed			0.67	3.505	0.546	
**CD86+ cells in SM**	**Equal variances assumed**	**4.16**	**0.111**	**-0.21**	**4**	**0.847**	**0.17**
	Equal variances not assumed			-0.21	2.346	0.854	
**CD11+ cells in SM**	**Equal variances assumed**	**2.77**	**0.171**	**-10.09**	**4**	**0.001**	**8.24**
	Equal variances not assumed			-10.09	2.482	0.004	
**CD206+ cells in SM**	**Equal variances assumed**	**0.00**	**0.973**	**-5.37**	**4**	**0.006**	**4.38**
	Equal variances not assumed			-5.37	3.999	0.006	
**HK of SM**	**Equal variances assumed**	**0.50**	**0.518**	**-0.16**	**4**	**0.884**	**0.13**
	Equal variances not assumed			-0.16	3.423	0.885	
**PFK of SM**	**Equal variances assumed**	**1.72**	**0.260**	**12.80**	**4**	**0,000**	**10.45**
	Equal variances not assumed			12.80	2.816	0.001	
**G6PD of SM**	**Equal variances assumed**	**0.85**	**0.408**	**-0.60**	**4**	**0.583**	**0.49**
	Equal variances not assumed			-0.60	3.492	0.587	
**H6PD of SM**	**Equal variances assumed**	**0.94**	**0.387**	**-3.70**	**4**	**0.021**	**3.02**
	Equal variances not assumed			-3.70	2.852	0.037	
**SM ER signal intensity**	**Equal variances assumed**	**0.82**	**0.415**	**0.02**	**4**	**0.983**	**0.02**
	Equal variances not assumed			0.02	3.516	0.983	
**SM 2-NBDG signal intensity**	**Equal variances assumed**	**7.93**	**0.048**	**-7.72**	**4**	**0.002**	**1.45**
	Equal variances not assumed			-7.72	2.161	0.013	
**% of ER voxels within 2-NBDG in SM**	**Equal variances assumed**	**4.81**	**0.093**	**-0.39**	**4**	**0.717**	**0.32**
	Equal variances not assumed			-0.39	2.577	0.727	
**% of 2-NBDG voxels within ER in SM**	**Equal variances assumed**	**0.00**	**0.982**	**-4.15**	**4**	**0.014**	**7.72**
	Equal variances not assumed			-4.15	4	0.014	
**NADPH in SM**	**Equal variances assumed**	**4.11**	**0.113**	**4.53**	**4**	**0.011**	**3.70**
	Equal variances not assumed			4.53	2.736	0.024	
**NADPH+NADP in SM**	**Equal variances assumed**	**0.00**	**0.995**	**-0.18**	**4**	**0.866**	**0.15**
	Equal variances not assumed			-0.18	4.000	0.866	
**GR activity of SM**	**Equal variances assumed**	**0.02**	**0.903**	**-3.99**	**4**	**0.016**	**3.26**
	Equal variances not assumed			-3.99	3.980	0.016	
**MDA content in SM**	**Equal variances assumed**	**3.42**	**0.138**	**-3.50**	**4**	**0.025**	**2.85**
	Equal variances not assumed			-3.50	2.069	0.069	
**H2DCFDA (MFI) in SM**	**Equal variances assumed**	**2.11**	**0.220**	**-10.35**	**4**	**0,000**	**8.45**
	Equal variances not assumed			-10.35	2.220	0.006	
**OCR of SM-mitochondria**	**Equal variances assumed**	**0.25**	**0.642**	**3.68**	**4**	**0.021**	**3.00**
	Equal variances not assumed			3.68	3.752	0.024	
**ATP synthesis of SM-mitochondria**	**Equal variances assumed**	**3.37**	**0.140**	**2.80**	**4**	**0.049**	**2.29**
	Equal variances not assumed			2.80	2.167	0.098	
**Complex I activity of SM-mitochondria**	**Equal variances assumed**	**2.53**	**0.187**	**-3.34**	**4**	**0.029**	**2.72**
	Equal variances not assumed			-3.34	2.520	0.057	
**Mfn2 expression in SM**	**Equal variances assumed**	**0.85**	**0.409**	**3.02**	**4**	**0.039**	**2.47**
	Equal variances not assumed			3.02	3.640	0.044	
**Drp1 expression in SM**	Equal variances assumed	8.11	0.047	-6.15	4	0.004	**5.02**
	**Equal variances not assumed**			**-6.15**	**2.228**	**0.019**	
**Calnexin expression in SM**	**Equal variances assumed**	**0.98**	**0.378**	**-0.05**	**4**	**0.964**	**0.04**
	Equal variances not assumed			-0.05	2.861	0.965	
**HK activity of Heart**	**Equal variances assumed**	**0.03**	**0.877**	**0.74**	**4**	**0.501**	**0.60**
	Equal variances not assumed			0.74	3.949	0.502	
**PFK activity of Heart**	**Equal variances assumed**	**4.34**	**0.106**	**-0.22**	**4**	**0.837**	**0.18**
	Equal variances not assumed			-0.22	2.590	0.843	
**G6PD activity of Heart**	**Equal variances assumed**	**0.78**	**0.428**	**0.92**	**4**	**0.411**	**0.75**
	Equal variances not assumed			0.92	3.467	0.419	
**H6PD activity of Heart**	**Equal variances assumed**	**0.88**	**0.403**	**-0.23**	**4**	**0.828**	**0.19**
	Equal variances not assumed			-0.23	3.383	0.83	
**GR activity of Heart**	Equal variances assumed	12.02	0.026	-0.93	4	0.408	**0.75**
	**Equal variances not assumed**			**-0.93**	**2.037**	**0.451**	

**MDA content in Heart**	**Equal variances assumed**	**4.41**	**0.104**	**1.03**	**4**	**0.362**	**0.84**
	Equal variances not assumed			1.03	2.374	0.397	
**H2DCFDA (MFI) in Heart**	**Equal variances assumed**	**1.17**	**0.340**	**-0.51**	**4**	**0.638**	**0.51**
	Equal variances not assumed			-0.51	3.496	0.642	
**OCR of Heart-mitochondria**	**Equal variances assumed**	**2.30**	**0.204**	**2.91**	**4**	**0.043**	**2.38**
	Equal variances not assumed			2.91	3.039	0.061	
**ATP synthesis of Heart-mitochondria**	Equal variances assumed	15.93	0.016	1.01	4	0.371	**0.82**
	**Equal variances not assumed**			**1.01**	**2**	**0.42**	
**Complex I activity of Heart-mitochondria**	**Equal variances assumed**	**1.93**	**0.238**	**-1.32**	**4**	**0.257**	**1.08**
	Equal variances not assumed			-1.32	2.948	0.28	
