# Supplementary material for: Mechanisms underlying the predictive power of high skeletal muscle uptake of FDG in amyotrophic lateral sclerosis
Source: EJNMMI Res. 2020 Jul 7;10:76. doi: 10.1186/s13550-020-00666-6 (PMC7340686; doi:10.1186/s13550-020-00666-6)
Supplement: Supplementary file 2 — Additional file 2: Supplementary Table 2. PET imaging data [file 13550_2020_666_MOESM2_ESM.pdf]

Supplementary Table 2: PET imaging data

		Mean	*SD*	*p*
Skeletal Muscle SUV	Control	0.33	0.03	0.042
	SOD1^{G93A}	0.44	0.09	
Myocardial SUV	Control	2.48	1.43	0.361
	SOD1^{G93A}	1.65	1.30	
Blood pool [kBq/ml]	Control	167.33	51.49	0.228
	SOD1^{G93A}	205.30	98.71	
Skeletal muscle FDG concentration [kBq/ml]	Control	51.54	5.87	0.043
	SOD1^{G93A}	88.14	33.52	
Myocardial FDG concentration [kBq/ml]	Control	369.53	204.69	0.922
	SOD1^{G93A}	388.56	365.65	
Skeletal Muscle VOI volume [µL]	Control	43.00	21.00	0.921
	SOD1^{G93A}	42.00	14.00	
Myocardial VOI volume [µL]	Control	10.00	6.00	0.666
	SOD1^{G93A}	9.00	3.00	
