# Supplementary material for: Mechanisms underlying the predictive power of high skeletal muscle uptake of FDG in amyotrophic lateral sclerosis
Source: EJNMMI Res. 2020 Jul 7;10:76. doi: 10.1186/s13550-020-00666-6 (PMC7340686; doi:10.1186/s13550-020-00666-6)
Supplement: Supplementary file 3 — Additional file 3. Supplementary Materials [file 13550_2020_666_MOESM3_ESM.docx]

**Mechanisms underlying the predictive power of high skeletal muscle uptake of FDG in Amyotrophic Lateral Sclerosis**

Cecilia Marini*, Vanessa Cossu*, Tiziana Bonifacino*, Matteo Bauckneht, Carola Torazza, Silvia Bruno, Patrizia Castellani, Silvia Ravera, Marco Milanese , Consuelo Venturi, Sebastiano Carlone, Patrizia Piccioli, Laura Emionite, Silvia Morbelli, Anna Maria Orengo, Maria Isabella Donegani, Alberto Miceli, Stefano Raffa, Stefano Marra, Alessio Signori, Katia Cortese, Federica Grillo, Roberto Fiocca, Giambattista Bonanno, Gianmario Sambuceti

*these authors contributed equally as first co-authors

**Supplementary Materials**

Table Legends.

*Supplementary Table 1.*

Table 1 displays statistical parameters of independent samples test. Levene’s test for equality of variances was used to test the homogeneity of variances of the two groups. When homogeneity wasn’t satisfied the Welch-Satterthwaite method was used to adjust the p-value for this assumption violation. Statistical significance was considered for p < 0.05. For each analysis, was reported the indexes of Levene's Test for Homoscedasticity (F value and statistical significance (Sig.)), the indexes of t-test for Equality of Means (t value, degrees of freedom (df) and statistical significance 2-tailed (Sig. 2-tailed)), and the effect size (Cohen's d). The considered values are reported in bold font.

*Supplementary Table 2.*

Table displays mean, standard deviation (SD) and p value of: i) skeletal muscle and myocardial standardized uptake value (SUV), ii) FDG concentration of blood pool, skeletal muscle and myocardium (expressed as kBq/ml) and iii) VOI values of both studied tissues in control and SOD1^G93A^ mice.

Supplementary Figure 1. Effect of SOD1^G93A^ in cardiac inflammatory infiltration.

Panels A, C and E report immunohistochemical representative images of CD86, CD11b and CD206 expression, in controls (green edge line) and SOD1^G93A^ (red edge line) myocardial slices. Panels B, D and F display the quantitative representation of the CD86^+^, CD11b^+^ and CD206^+^ signal, expressed as percent of the image field occupied by the specific cell staining. Data are expressed as mean ± SD, n samples=3 for each group. Student t test for unpaired data was used for statistical evaluation.
