# Supplementary figures and images for: Mechanisms underlying the predictive power of high skeletal muscle uptake of FDG in amyotrophic lateral sclerosis
Source: EJNMMI Res. 2020 Jul 7;10:76. doi: 10.1186/s13550-020-00666-6 (PMC7340686; doi:10.1186/s13550-020-00666-6)

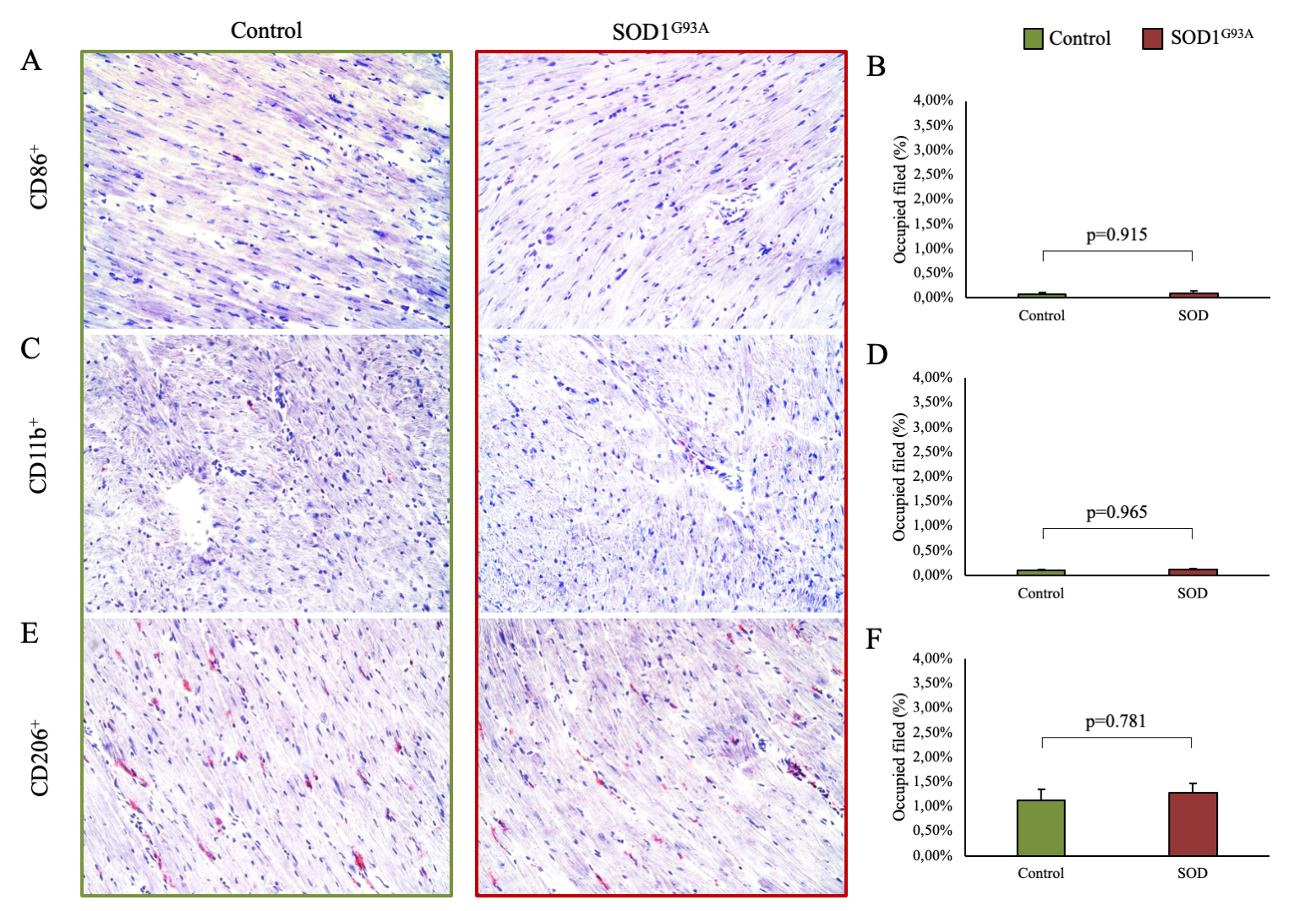

Supplement: Supplementary file 4 — Additional file 4. Suppl Figure [file 13550_2020_666_MOESM4_ESM.tiff]
